# Assessment of Various Archwire Materials and Their Impact on Orthodontic Treatment Outcomes

**DOI:** 10.7759/cureus.69667

**Published:** 2024-09-18

**Authors:** Bharathi V S, Ankur Kaul, Anurag Tiwari, Subhi Aliya, Apna Yadav, Trinanjali Bera, Preet Kaur Makkad

**Affiliations:** 1 Orthodontics and Dentofacial Orthopedics, RajaRajeswari Dental College & Hospital, Bangalore, IND; 2 Orthodontics, Auburn Dental Group, Auburn, USA; 3 Orthodontics and Dentofacial Orthopedics, National Institute of Medical Sciences (NIMS) Dental College and Hospital, NIMS University, Jaipur, IND; 4 Orthodontics, Balaghat Dental Clinic, Balaghat, IND; 5 Oral and Maxillofacial Surgery, Faculty of Dental Sciences, Shree Guru Gobind Singh Tricentenary (SGT) University, Gurugram, IND; 6 Dentistry, Kalinga Institute of Dental Sciences, Kalinga Institute of Industrial Technology (KIIT) Deemed to be University, Bhubaneswar, IND; 7 Dentistry, Gyan Dental Clinic, Bilaspur, IND

**Keywords:** archwire materials, frictional properties, mechanical properties, modulus of elasticity, orthodontics, surface characteristics, surface roughness, tensile strength, yield strength

## Abstract

Aim

Orthodontic treatment relies heavily on the mechanical properties and surface characteristics of archwire materials to achieve optimal outcomes. This study aimed to comprehensively evaluate the mechanical properties, including tensile strength, yield strength, and modulus of elasticity, as well as the surface characteristics, such as surface roughness and frictional properties, of different archwire materials.

Methods

Four types of archwire materials, stainless steel, nickel-titanium (NiTi), beta-titanium, and esthetic archwires, were subjected to mechanical testing and surface analysis, with 31 in each group. Tensile testing was conducted to determine the maximum tensile strength, yield strength, and elastic modulus of each material. Surface roughness analysis was performed using profilometry techniques, and frictional properties were evaluated using an orthodontic friction testing apparatus.

Results

Stainless steel exhibited the highest tensile strength (900 N), followed by beta-titanium (850 N), NiTi (800 N), and esthetic archwire (750 N). Stainless steel also demonstrated the highest yield strength (780 N), followed by beta-titanium (740 N), NiTi (710 N), and esthetic archwire (650 N). The modulus of elasticity was the highest for stainless steel (200 GPa), followed by beta-titanium (170 GPa), NiTi (150 GPa), and esthetic archwires (120 GPa). Surface roughness was lowest in stainless steel archwires (mean Ra value of 0.25 µm), leading to reduced frictional resistance, whereas esthetic archwires exhibited the highest surface roughness (mean Ra value of 0.40 µm) and frictional forces. Significant differences in the mechanical properties and surface characteristics were observed among the materials (p < 0.05).

Conclusions

The choice of archwire material significantly influences orthodontic treatment outcomes by affecting the efficiency and effectiveness of tooth movement. Stainless steel and beta-titanium wires are ideal for high-stress applications, providing the robust mechanical strength necessary for complex movements. In contrast, NiTi wires, with their superelasticity, offer consistent and gentle forces, enhancing patient comfort and accelerating the alignment phase. Esthetic archwires, while visually appealing, often compromise mechanical performance, potentially prolonging treatment duration.

## Introduction

Orthodontic treatment aims to correct dental and skeletal discrepancies and improve both function and aesthetics [1]. Central to orthodontic therapy is the use of archwires, which play a crucial role in applying the necessary forces to move the teeth into their desired positions. The choice of archwire material significantly affects treatment efficiency, patient comfort, and overall treatment outcomes [2]. Various materials, including stainless steel, nickel-titanium (NiTi), beta-titanium, and esthetic archwires, offer distinct mechanical properties and surface characteristics that influence their performance in clinical applications [3]. Stainless steel is a staple in orthodontics due to its high strength, stiffness, and corrosion resistance. Its mechanical properties enable it to withstand substantial forces without permanent deformation, making it ideal for the later stages of treatment, where precise tooth positioning and torque control are crucial. The high modulus of elasticity of stainless steel archwires provides the rigidity necessary for effective torque control and precise tooth positioning, ensuring stable and accurate results [4,5].

NiTi archwires have revolutionized orthodontic treatment due to their superelastic properties and shape memory effects. Their high tensile strength ensures durability and the ability to withstand significant forces without breaking, essential for sustained tooth movement. The high yield strength allows these archwires to deform significantly while returning to their original shape, applying consistent and gentle forces for gradual and controlled tooth realignment. Additionally, a lower modulus of elasticity provides flexibility, enabling effective engagement with brackets and reducing the risk of damage to teeth and surrounding structures, thereby enhancing patient comfort. These characteristics allow NiTi archwires to deliver continuous, gentle forces over a wide range of deflections, facilitating large tooth movements with minimal discomfort for patients [6,7]. The superelasticity of NiTi is particularly beneficial during the alignment and leveling phases of the treatment, where flexibility and consistent force application are paramount [8]. Despite their advantages, NiTi archwires typically have lower tensile and yield strengths than stainless steel, which may limit their application in later treatment stages [9]. Beta-titanium, or titanium-molybdenum alloy, offers a balance between the stiffness of stainless steel and the flexibility of NiTi. It exhibits moderate tensile strength and yield strength, along with a lower modulus of elasticity than stainless steel [10]. This combination of properties makes beta-titanium a versatile material suitable for various clinical scenarios, including situations requiring both resilience and precise control of tooth movement [11].

Esthetic archwires, which include coated metal and composite materials, cater to the increasing demand for aesthetically pleasing orthodontic appliances. These archwires are designed to blend with natural tooth colors, making them less noticeable [3,12]. However, the coatings or materials used to achieve this esthetic appearance often compromise the mechanical properties and increase surface roughness, potentially affecting the efficiency of tooth movement [13]. The mechanical properties of archwires, such as their tensile strength, yield strength, and modulus of elasticity, are critical determinants of their performance in orthodontic applications. Tensile strength measures the maximum stress that an archwire can withstand while being stretched before breaking, whereas yield strength indicates the stress level at which the archwire begins to deform permanently [14]. The modulus of elasticity reflects the stiffness of the material, influencing its ability to maintain the desired arch form under a load [14].

In addition to the mechanical properties, the surface characteristics of archwires, including surface roughness and frictional properties, play a significant role in the efficiency of orthodontic treatment. Surface roughness affects the interaction between archwire and orthodontic brackets, with smoother surfaces typically resulting in lower frictional forces and more efficient tooth movement. High frictional resistance can impede sliding mechanics, prolong treatment time, and potentially compromise outcomes [15].

This study aimed to comprehensively assess the mechanical properties and surface characteristics of different archwire materials and their influence on orthodontic treatment efficiency. By comparing stainless steel, NiTi, beta-titanium, and esthetic archwires, we sought to provide valuable insights into their clinical performance and guide material selection to optimize treatment outcomes. Understanding the strengths and limitations of each material will enable orthodontists to tailor their use to specific clinical scenarios, thereby enhancing the effectiveness and efficiency of orthodontic therapy.

## Materials and methods

Sample selection

A total of 124 samples of various archwire materials commonly used in orthodontic treatment were selected for this study. These include stainless steel, NiTi, beta-titanium, and esthetic archwires. The samples were sourced from reputable manufacturers to ensure consistency and reliability during the evaluation. The sample size for this study was determined through a power analysis to ensure adequate power to detect significant differences in mechanical and surface properties among various archwire materials. Based on an expected effect size derived from previous studies, a significance level (α) of 0.05, and a desired power (1-β) of 0.80, it was calculated that at least 10 samples per group were required. To enhance the study’s robustness and account for potential variability, the sample size was increased to 124 samples, with 31 samples per each of the four groups: stainless steel, NiTi, beta-titanium, and esthetic archwires. This ensures higher precision and reliability in evaluating the mechanical behavior and surface characteristics of the archwire materials under investigation.

Material characterization

During the process of material characterization, each archwire material underwent a thorough assessment to ascertain both its mechanical properties and surface characteristics. This involved a multifaceted approach, encompassing various testing methodologies. Tensile testing, a fundamental procedure in material science, was performed using a universal testing machine, specifically the Instron 5965, which was meticulously calibrated to adhere to the precise specifications outlined by the manufacturer. This ensured an accurate and consistent measurement of the mechanical behavior of the archwire samples under tensile loading conditions. The key parameters evaluated during tensile testing included the tensile strength, which signifies the maximum stress that a material can withstand before fracturing; the yield strength, which indicates the stress at which the material begins to exhibit permanent deformation; and the modulus of elasticity, which reflects the stiffness of the material and its resistance to deformation under tensile stress. Surface roughness analysis was conducted using profilometry techniques, which provided detailed insights into the topographical features of archwire sample surfaces. Profilometry allows for the precise quantification of surface irregularities and deviations from an idealized surface profile. The mean surface roughness (Ra) was determined as a quantitative measure of the average deviation of the surface profile from the mean line over a specified sampling length. This parameter is particularly relevant in orthodontic applications because it can influence the frictional forces and interactions between the archwire and orthodontic brackets. In addition, the frictional properties were evaluated using a specialized orthodontic friction testing apparatus. This apparatus simulated the sliding movement between the archwire and orthodontic brackets under controlled conditions, allowing measurement of the coefficient of friction for each archwire material. The coefficient of friction quantifies the resistance encountered during the sliding motion and is crucial for assessing the efficiency of orthodontic treatment mechanics. Understanding the frictional behavior of different archwire materials is essential for optimizing treatment protocols and achieving desirable tooth movement outcomes.

Sample preparation

During the sample preparation phase, meticulous attention was devoted to ensuring the consistency and integrity of the archwire samples prior to testing. Adhering to standardized dimensions and protocols, each sample was meticulously prepared to meet the specific criteria essential for reliable and reproducible test results. The first step in sample preparation involved precisely cutting the archwire material to the predetermined dimensions specified by the testing protocol. This was executed with the utmost precision, using specialized cutting tools to ensure uniformity in sample size across all specimens. By strictly adhering to standardized dimensions, any potential sources of variability stemming from discrepancies in sample size were minimized, thereby enhancing the reliability and validity of the experimental data. Care was taken to shape the archwire samples according to the designated geometry outlined in the testing protocol. Whether shaping the samples into specific configurations or ensuring consistent cross-sectional profiles, meticulous attention to detail was maintained throughout the sample preparation process. This meticulous approach helped minimize variations in the sample geometry, which could otherwise introduce confounding factors during testing and compromise the accuracy of the results. Furthermore, each sample was handled with the utmost care to prevent any inadvertent damage or alteration of its surface characteristics. Special precautions were taken to avoid introducing contaminants or imperfections that could potentially influence the evaluated material properties. This involved handling the samples with clean, gloved hands and employing appropriate storage techniques to safeguard them against environmental factors that could compromise sample integrity.

Tensile testing

During the tensile testing process, each archwire sample underwent a comprehensive evaluation of its mechanical properties under controlled conditions. To ensure accuracy and consistency, the samples were securely clamped within the grips of a universal testing machine, with careful attention paid to maintaining a uniform grip-to-grip distance. This consistent clamping configuration minimized the potential sources of variability in the test results, ensuring reliable comparisons between different materials. The tensile force was then applied to the samples at a constant rate, following a predefined loading protocol. This gradual application of force allowed for a systematic assessment of the response of the samples to increasing levels of stress. The testing continued until failure occurred, characterized by the point at which the archwire sample fractured or experienced irreversible deformation. Throughout the testing process, the load-displacement behavior exhibited by each sample was closely monitored and recorded in real time.

**Figure 1:**
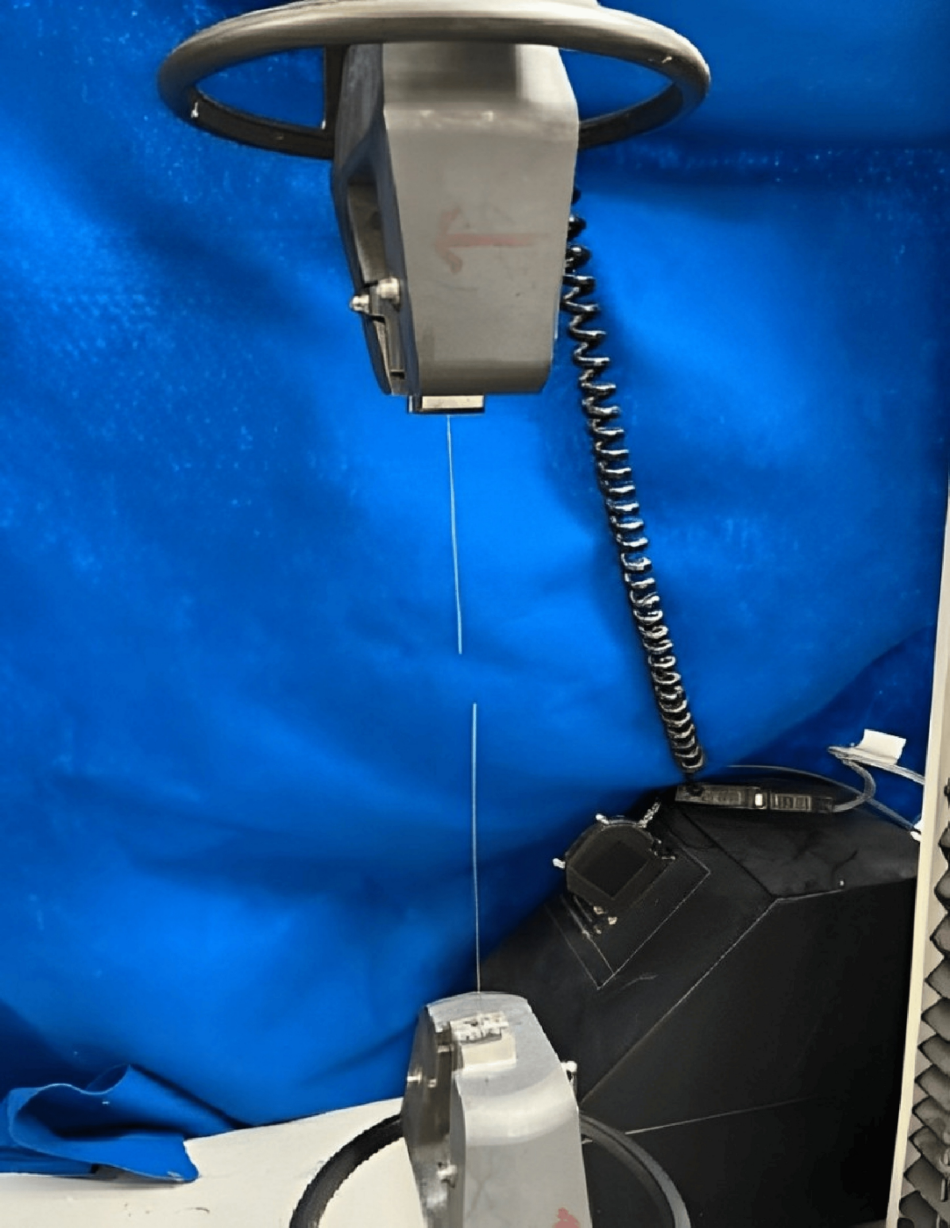
Tensile strength testing on the universal testing machine

Upon reaching the point of failure, several key mechanical properties were determined for each archwire. The maximum tensile strength, which represents the peak load sustained by the material before failure, was recorded. This parameter serves as a critical indicator of the structural integrity and load-bearing capacity of the material. Additionally, the yield strength, indicative of the stress at which the material begins to exhibit permanent deformation, was identified from the load-displacement curve. Furthermore, the modulus of elasticity, calculated based on the slope of the linear portion of the curve, provides insights into the stiffness of the material and resistance to deformation under tensile stress (Figure 1).

Surface roughness analysis

**Figure 2:**
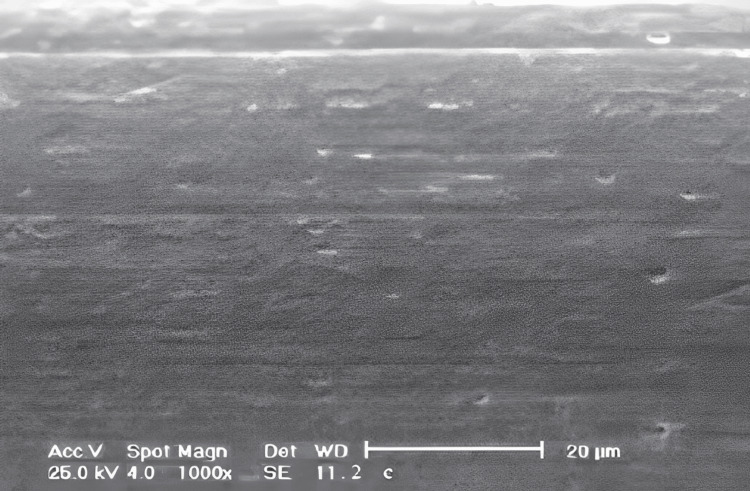
Surface of the archwire under scanning electron microscopy to check for surface roughness

Surface roughness analysis involved a detailed examination of the topographical features of the archwire sample surfaces to quantify their roughness characteristics. Profilometry, a precise measurement technique, was employed to obtain accurate data regarding the surface roughness. Multiple profilometric scans were conducted across each sample to ensure comprehensive coverage and obtain representative measurements of surface roughness. This involved systematically moving the profilometer probe across the sample surface while recording height variations with a high spatial resolution. By capturing data from multiple scan lines, a detailed profile of the surface topography was generated, allowing for a comprehensive assessment of surface roughness. The mean surface roughness (Ra) was calculated for each archwire material based on profilometric data. Ra represents the average deviation of the surface profile from the mean line for a specified sampling length. This parameter serves as a quantitative measure of the surface roughness, providing valuable information about the texture and irregularities present on the archwire surfaces (Figure 2).

Frictional testing

**Figure 3:**
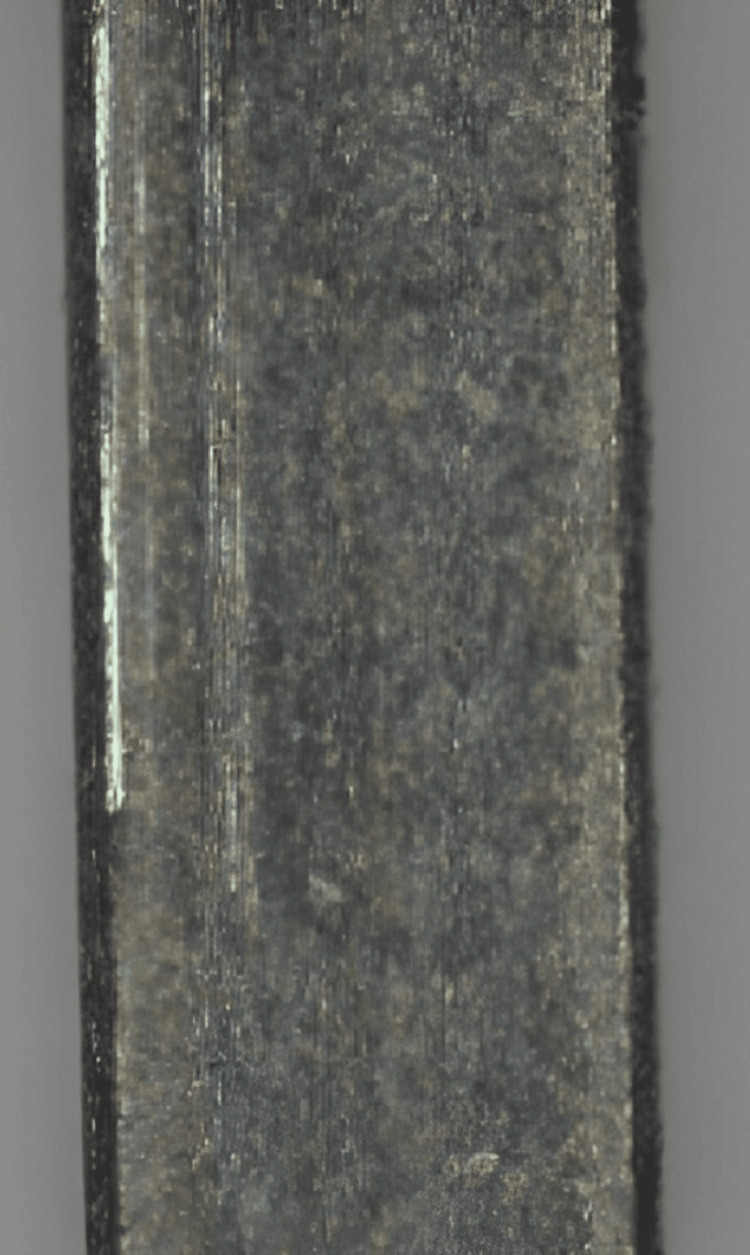
Frictional test of the orthodontic archwire on the machine

Frictional testing was conducted to assess the interaction between archwire samples and orthodontic brackets under simulated sliding movements. This evaluation provided insights into the frictional properties of materials, which are critical considerations in orthodontic treatment planning and mechanics. The archwire samples were mounted within the orthodontic friction testing apparatus, and standardized conditions were established for frictional testing. This ensured the consistency and reproducibility of the experimental setup, facilitating accurate comparisons between different materials. During testing, the samples were subjected to simulated sliding movements within the testing apparatus to mimic the dynamic conditions encountered during orthodontic tooth movement. The resistance encountered during these movements was measured, allowing the determination of the coefficient of friction for each material. The coefficient of friction quantifies the resistance to motion between two surfaces in contact and is a key parameter for assessing the efficiency of orthodontic treatment mechanics. By evaluating the frictional properties of archwire materials, valuable insights have been gained into their performance characteristics and suitability for various clinical applications (Figure 3).

Data analysis

IBM SPSS Statistics for Windows, Version 24.0 (Released 2016; IBM Corp., Armonk, NY, USA) was used for data analysis. Descriptive statistics, including mean, standard deviation, and minimum and maximum values, were calculated for the mechanical properties and surface characteristics of each archwire material. A comparative analysis was conducted to identify any significant differences in material properties among the different archwires using appropriate statistical tests such as one-way ANOVA or nonparametric equivalents. Statistical significance was set at p < 0.05 to determine the significance of observed differences.

## Results

Table 1 summarizes the tensile strengths of different archwire materials.

**Table 1:** Tensile strength of archwire materials NiTi, nickel-titanium

Archwire material	Mean (N)	Standard deviation (N)	Minimum (N)	Maximum (N)
Stainless steel	900	15.5	870	925
NiTi	800	20.3	765	835
Beta-titanium	850	17.8	820	875
Esthetic archwire	750	18.2	715	785

The mean tensile strength values for stainless steel, 800 N for NiTi, 850 N for beta-titanium, and esthetic archwires were 900, 800, 850, and 750 N, respectively. The stainless steel had the highest tensile strength (900 N, SD = 15.5 N), indicating its superior load-bearing capacity. Esthetic archwires had the lowest tensile strength (750 N, SD = 18.2 N), suggesting that they may be less suitable for high-stress applications. The ANOVA results show a significant difference in tensile strength among the materials (F = 25.76, p < 0.001), confirming that the variations in tensile strength were statistically significant. Therefore, stainless steel and beta-titanium are preferable for applications that require high mechanical strength.

Table 2 displays the yield strength values of 780, 710, 740, and 650 N for stainless steel, NiTi, 740 N for beta-titanium, and 650 N for esthetic archwires, respectively.

**Table 2:** Yield strength of archwire materials NiTi, nickel-titanium

Archwire material	Mean (N)	Standard deviation (N)	Minimum (N)	Maximum (N)
Stainless steel	780	12.4	755	800
NiTi	710	15.6	680	735
Beta-titanium	740	14.2	715	765
Esthetic archwire	650	16.5	620	675

The stainless steel demonstrated the highest yield strength (780 N, SD = 12.4 N), indicating its high resistance to permanent deformation. The esthetic archwires had the lowest yield strength (650 N, SD = 16.5 N). The ANOVA results (F = 18.34, p < 0.001) confirm that the differences in yield strength among the materials are statistically significant. Hence, stainless steel and beta-titanium are more resistant to deformation than NiTi and esthetic archwire.

Table 3 lists the modulus of elasticity for each archwire material: 200 GPa for stainless steel, 150 GPa for NiTi, 170 GPa for beta-titanium, and 120 GPa for esthetic archwires.

**Table 3:** Modulus of elasticity of archwire materials NiTi, nickel-titanium

Archwire material	Mean (GPa)	Standard deviation (GPa)	Minimum (GPa)	Maximum (GPa)
Stainless steel	200	3.1	195	205
NiTi	150	2.7	145	155
Beta-titanium	170	2.9	165	175
Esthetic archwire	120	3.3	115	125

The stainless steel exhibited the highest modulus of elasticity (200 GPa, SD = 3.1 GPa), reflecting its stiffness. Esthetic archwires have the lowest modulus of elasticity (120 GPa, SD = 3.3 GPa), indicating that they are more flexible. The ANOVA results (F = 22.45, p < 0.001) revealed significant differences in the modulus of elasticity among the materials. These findings suggest that stainless steel and beta-titanium provide the necessary stiffness for certain orthodontic applications, whereas esthetic archwires might be better suited for cases that require more flexibility.

Surface roughness analysis in Table 4 shows mean Ra values of 0.25 µm for stainless steel, 0.35 µm for NiTi, 0.30 µm for beta-titanium, and 0.40 µm for esthetic archwires.

**Table 4:** Surface roughness (Ra) of archwire materials NiTi, nickel-titanium

Archwire material	Mean (µm)	Standard deviation (µm)	Minimum (µm)	Maximum (µm)
Stainless steel	0.25	0.05	0.2	0.3
NiTi	0.35	0.07	0.28	0.42
Beta-titanium	0.3	0.06	0.24	0.36
Esthetic archwire	0.4	0.08	0.32	0.48

Esthetic archwires had the highest surface roughness (0.40 µm, SD = 0.08 µm), which may impact frictional properties and bracket interaction. Stainless steel had the smoothest surface (0.25 µm, SD = 0.05 µm). The significant ANOVA results (F = 15.29, p < 0.001) confirmed the statistical significance of the differences in surface roughness. These differences highlight the importance of the surface characteristics in determining the clinical performance of archwire materials.

Table 5 reports the coefficient of friction for each archwire material: 0.25 for stainless steel, 0.30 for NiTi, 0.28 for beta-titanium, and 0.35 for esthetic archwires.

**Table 5:** Coefficient of friction of archwire materials NiTi, nickel-titanium

Archwire material	Mean	Standard deviation	Minimum	Maximum
Stainless steel	0.25	0.02	0.23	0.27
NiTi	0.3	0.03	0.27	0.33
Beta-titanium	0.28	0.02	0.26	0.3
Esthetic archwire	0.35	0.04	0.31	0.39

Esthetic archwires exhibited the highest coefficient of friction (0.35, SD = 0.04), indicating greater resistance during sliding movements. Stainless steel had the lowest coefficient of friction (0.25, SD = 0.02). The ANOVA results (F = 20.62, p < 0.001) reveal significant differences in the coefficient of friction among the materials, emphasizing the impact of material properties on frictional resistance in orthodontic treatment (Tables 6, 7).

**Table 6:** ANOVA results for mechanical properties

Property	F-value	p-value
Tensile strength	25.76	<0.001
Yield strength	18.34	<0.001
Modulus of elasticity	22.45	<0.001

**Table 7:** ANOVA results for surface characteristics

Property	F-value	p-value
Surface roughness (Ra)	15.29	<0.001
Coefficient of friction	20.62	<0.001

## Discussion

This study aimed to assess the mechanical properties and surface characteristics of different archwire materials and their potential impact on orthodontic treatment efficiency. Our findings revealed significant differences in the tensile strength, yield strength, modulus of elasticity, surface roughness, and coefficient of friction among the materials tested. These differences have important clinical implications for the selection of archwires in orthodontic practice. Stainless steel archwires demonstrated the highest tensile strength and yield strength among the materials tested. This aligns with previous research highlighting the robust mechanical properties of stainless steel, making it the preferred choice for high-stress applications in orthodontics [4]. The high tensile and yield strengths suggest that multi-stranded stainless-steel archwires can withstand significant loads without permanent deformation, providing reliable performance in the aligning and leveling phases of orthodontic treatment [2].

Beta-titanium archwires also show favorable mechanical properties, with tensile strength and yield strength values close to those of stainless steel. This is consistent with earlier studies that have recognized the utility of beta-titanium in situations where both strength and flexibility are required [16]. The intermediate modulus of elasticity observed in beta-titanium further supports its use in clinical scenarios that demand a balance between rigidity and resilience [16]. NiTi archwires exhibited lower tensile and yield strengths than stainless steel and beta-titanium but were characterized by a unique superelastic property and shape memory effect. These properties enable NiTi archwires to deliver continuous and gentle forces over a range of tooth movements, making them particularly effective during the initial stages of treatment, when large tooth movements are required [2]. The lower modulus of elasticity of NiTi contributes to its flexibility, allowing it to engage multiple teeth without causing excessive discomfort to patients [17]. Esthetic archwires, while aesthetically pleasing, exhibited the lowest mechanical strength among the tested materials. The reduced tensile and yield strengths of esthetic archwires suggest that they are more prone to deformation under stress, potentially limiting their use to less demanding phases of treatment or in cases where esthetics are a primary concern [18].

Surface roughness and frictional properties are critical factors that influence the efficiency of orthodontic treatment. The surface roughness of the archwires affects the amount of friction encountered between the wire and brackets, which in turn affects the efficiency of tooth movement. Our study found that stainless-steel archwires had the smoothest surface, followed by beta-titanium, NiTi, and esthetic archwires. The smooth surface of stainless steel archwires, as indicated by the low mean Ra value (0.25 µm), contributes to reduced frictional forces during sliding mechanics. This finding corroborates the existing literature that has consistently shown that stainless steel exhibits low frictional resistance owing to its polished surface finish. Reduced friction is advantageous in the later stages of treatment when precise tooth movements are critical [19].

The beta-titanium archwires displayed intermediate surface roughness values. Although rougher than stainless steel, beta-titanium still offers relatively low friction, which is beneficial in clinical situations requiring moderate control over tooth movement [16]. The surface properties of beta-titanium, combined with its mechanical resilience, make it a versatile material for orthodontic treatment [16]. NiTi archwires exhibited a higher surface roughness than stainless steel and beta-titanium. The increased roughness of NiTi wires (mean Ra value of 0.35 µm) can contribute to higher frictional resistance, which may affect the efficiency of tooth movement during sliding mechanics [20]. However, the unique superelastic properties of NiTi can compensate for this by providing consistent forces that facilitate effective tooth movement despite higher friction [20]. Esthetic archwires had the highest surface roughness (mean Ra value of 0.40 µm), which aligns with a previous study noting the inherent roughness of coated or composite materials used for esthetic purposes [21]. The high friction associated with esthetic archwires can slow the tooth movement process, potentially extending the duration of treatment. Therefore, while esthetic archwires are appealing for their visual advantages, their use should be carefully considered in the context of their frictional characteristics.

The coefficient of friction results mirrored the surface roughness findings, with stainless steel exhibiting the lowest coefficient of friction (0.25), followed by beta-titanium (0.28), NiTi (0.30), and esthetic archwire (0.35). The lower frictional forces observed with stainless steel are advantageous for efficient tooth movement, particularly in the final stages of orthodontic treatment where precise adjustments are necessary [22]. The significant differences in the mechanical and surface properties among the tested archwire materials underscore the importance of selecting the appropriate archwire based on the specific requirements of each stage of orthodontic treatment. Stainless steel wires are suitable for high-stress applications and scenarios requiring precise control, owing to their high mechanical strength and low frictional resistance. NiTi archwires, owing to their superelasticity and shape memory, are ideal for the initial alignment and leveling stages, offering consistent forces that facilitate large tooth movements while minimizing patient discomfort. Esthetic archwires, which are beneficial for patients with a high demand for aesthetics, should be used judiciously, given their lower mechanical strength and higher frictional resistance. Their application might be best limited to less demanding phases of treatment or as an auxiliary wire to enhance visual appeal without compromising overall treatment efficiency [3].

The findings of this study align closely with existing literature, reinforcing the notion that stainless steel archwires remain the gold standard in orthodontic treatment due to their superior mechanical properties and low frictional resistance. High tensile strength and a low coefficient of friction in stainless steel make it ideal for high-stress and precision-driven phases of treatment. Meanwhile, beta-titanium’s balance of strength and flexibility has been consistently supported in clinical settings where moderate force application and resilience are critical. The unique properties of NiTi, particularly its superelasticity and shape memory, have been widely recognized in early-stage treatments requiring consistent force over a broad range of tooth movements. However, the higher friction associated with esthetic archwires, despite their visual appeal, presents a significant challenge, indicating that these wires may be better suited for less demanding treatment phases where aesthetics are prioritized over mechanical performance. These comparisons underscore the importance of selecting archwires based on the specific mechanical and clinical demands of each treatment phase, highlighting a tailored approach to orthodontic care.

Limitations and future research

Although this study provides valuable insights into the properties of various archwire materials, it has several limitations. The in vitro nature of this study means that the results might not fully replicate the clinical environment in which factors such as oral fluids, temperature variations, and patient-specific characteristics can influence the performance of archwires. Future research should include in vivo studies to validate these findings and explore the long-term effects of different archwire materials on treatment outcomes. Moreover, this study focuses on a limited number of archwire materials. Expanding the range of materials tested, including new alloys and composite materials, could provide a more comprehensive understanding of the options available to orthodontists. Additionally, the influence of different bracket systems on the frictional properties of archwires should be investigated because the interaction between the wire and bracket plays a crucial role in the overall efficiency of orthodontic treatment.

## Conclusions

This study highlighted the significant variability in the mechanical properties and surface characteristics of different archwire materials. NiTi archwires, with their unique superelastic properties, are well suited for the initial stages of treatment, requiring gentle, consistent forces. Esthetic archwires, while visually appealing, present challenges owing to their lower mechanical strength and higher frictional resistance. These findings underscore the importance of material selection in optimizing orthodontic treatment efficiency and outcomes. Future research should focus on in vivo studies and explore a broader range of materials to further enhance clinical decision-making in orthodontics.
